# Economic Burden and Treatment Patterns Among Patients Diagnosed With Vitiligo in Japan: An Observational Retrospective Cohort Study

**DOI:** 10.1111/1346-8138.70458

**Published:** 2026-08-27

**Authors:** Naoki Oiso, Kent A. Hanson, Ahmed M. Soliman, Chao Li, Manabu Taura, Daniel Mercer, Andrew Rava, Danielle Bynoe, Haiyan Sun, Tamio Suzuki

**Affiliations:** ^1^ Department of Dermatology Kindai University Nara Hospital Ikoma Japan; ^2^ AbbVie Inc. North Chicago Illinois USA; ^3^ AbbVie GK Tokyo Japan; ^4^ Genesis Research Group Hoboken New Jersey USA; ^5^ Department of Dermatology Yamagata University Yamagata Japan; ^6^ Omoriekimae Hifuka Clinic Tokyo Japan

**Keywords:** claims analysis, economic burden, hypopigmentation, prescribing patterns, vitiligo

## Abstract

Vitiligo, a common and chronic depigmentation disorder, significantly impacts patients' quality of life and exerts a substantial economic burden. The deleterious impact of vitiligo can be exacerbated by comorbid conditions, including psychiatric and autoimmune conditions. Treatment may vary across geographic regions, and there is sparse evidence of treatment patterns for vitiligo in Japan. Evaluations of healthcare costs are also needed to help underline unmet needs, including data on the influence of comorbid conditions on costs. Here we report results of a retrospective cohort analysis using claims data from the Japan Medical Data Center database to evaluate the economic burden and treatment patterns among patients with vitiligo in Japan. Primary study outcomes were costs and healthcare resource utilization (HCRU) 12 months post‐index (diagnosis date) for patients with vitiligo versus matched controls, based on inpatient, outpatient, and pharmacy claims. In total, 28 800 patients were included (4800 in the vitiligo cohort; 24 000 matched controls). Patients with vitiligo had significantly greater all‐cause healthcare costs (¥84 766 vs. ¥55 011, *p* < 0.0001) and HCRU than matched controls, primarily driven by outpatient, dermatology‐, and vitiligo‐related procedure costs. In patients with vitiligo, comorbid psychiatric and autoimmune conditions significantly increased all‐cause healthcare costs versus no comorbid condition (¥193 468 vs. ¥78 313 and ¥185 179 vs. ¥77 184, respectively; both *p* < 0.0001). Most patients (67.5%) did not receive treatment for vitiligo in the 12 months following diagnosis. Of those who did, 76.0% received only phototherapy. Only 2.2% received second‐line treatment. Overall, Japanese patients with vitiligo face substantially higher healthcare costs and resource utilization than those without vitiligo, with burden further increased by psychiatric and autoimmune comorbidities; notably, most remained untreated in the year after diagnosis, and treated patients primarily received phototherapy.

## 1 Introduction

Vitiligo is a common and chronic depigmentation disorder that affects approximately 0.5% of the population of Japan [1]. Vitiligo is characterized by a partial or almost complete absence of functioning melanocytes, which manifests as skin depigmentation across the body [2]. Currently, vitiligo has no cure and is considered one of the most difficult‐to‐treat dermatological conditions [3, 4], even though many potential therapeutic approaches are available. Most treatments aim to encourage re‐pigmentation and include topical immunomodulators, corticosteroids, phototherapy, and pigment cell transplants/grafts. In severe and/or persistent cases, depigmentation of the remaining skin is also considered [4, 5, 6].

While vitiligo does not directly cause pain and seldom causes itching, it is associated with substantial health‐related quality‐of‐life burden and a higher burden of anxiety, depression, and other autoimmune diseases [1, 4, 7], which may contribute to increased economic and treatment burden for patients, payers, and the healthcare system [3, 8, 9, 10]. Recent large‐scale US database claims studies have shown that patients with vitiligo experience significantly higher annual healthcare costs and healthcare resource utilization (HCRU) compared with matched controls, with evidence suggesting that this burden may be amplified by the high prevalence of autoimmune and psychiatric comorbidities [3, 11]. Real‐world treatment patterns also suggest substantial unmet need, with many patients receiving no treatment following diagnosis and limited treatment escalation among those who are treated [4]. However, no such studies have been conducted in Japan.

Given the substantial variations between countries and global regions on treatment availability and treatment practices, it is important that the economic burden and prescribing practices for vitiligo in Japan are established. These data can inform resource allocation, evaluate the costs and benefits of treatments, and improve the efficiency of health service utilization for current and future clinical practice.

Here we present the results of a retrospective cohort study in Japan with three primary aims: (1) to compare direct healthcare costs and HCRU between vitiligo cases and matched non‐vitiligo controls; (2) to compare direct healthcare costs and HCRU among patients with vitiligo who have psychiatric or autoimmune comorbidities versus those without; and (3) to describe treatment patterns within the vitiligo cohort.

## 2 Methods

### 2.1 Study Design and Data Source

This was a retrospective cohort analysis using claims data from the Japan Medical Data Center (JMDC) database to evaluate treatment patterns and the economic burden among patients with vitiligo in Japan. The JMDC manages claims data in partnership with Japanese payers (the employer Health Insurance Associations), covers approximately 3 million people of working age per year (aged < 65 years), and has a cumulative dataset of around 17 million patients [12]. Claims data collected by the JMDC include information on patient enrollment, demographics, diagnoses, inpatient and outpatient claims, and dispensing receipts, as well as associated costs for each visit [12].

The study was conducted in accordance with the International Society for Pharmacoeconomics and Outcomes Research Good Practices for Outcomes Research and all applicable country‐specific requirements. Because this was a retrospective study using only anonymized data from the JMDC, no Ethics Committee/Institutional Review Board approval or informed patient consent was required.

### 2.2 Patients

Patients eligible for the study were aged at least 12 years with a new diagnosis of vitiligo according to the 10th revision of the International Classification of Diseases (ICD‐10; codes L80.x, H02.73, or N90.89) recorded on a nondiagnostic medical claim between October 1, 2016 and October 31, 2023. The date of the first diagnosis was considered the index date. Patients also had to have at least 12 months of continuous medical/pharmacy enrollment both pre‐ and post‐index. Patients were excluded if they had evidence of vitiligo‐related treatment or diagnosis during the 12‐month pre‐index period.

Non‐vitiligo controls (i.e., people with no diagnosis of vitiligo during the whole study period [October 1, 2015 to October 31, 2024]) were matched to vitiligo cases in a 5:1 ratio by age, sex, and geographical location. Index dates for controls were assigned randomly, with a date distribution similar to that of the vitiligo cohort.

### 2.3 Outcomes

The primary study outcomes were all‐cause HCRU and associated direct costs in the 12‐month post‐index period (including the index date) period in patients with vitiligo versus matched non‐vitiligo controls. HCRU included inpatient admissions, emergency department visits, outpatient office visits, dermatology visits, outpatient prescriptions, and vitiligo‐related treatments and procedures. Vitiligo‐related treatments included topical/oral corticosteroids, systemic immunosuppressants, topical calcineurin inhibitors, topical vitamin D, and depigmentation treatments (e.g., monobenzone); vitiligo‐related procedures included phototherapy (e.g., ultraviolet‐light treatment, laser treatment, excimer laser therapy), photochemotherapy, dermabrasion, and epidermal skin grafts. Given the increased incidence and potential impact of comorbidities in patients with vitiligo, all‐cause and vitiligo‐related HCRU and associated direct costs among vitiligo cases were stratified by the presence of psychiatric or autoimmune (including immune‐mediated inflammatory) conditions. Vitiligo‐related HCRU was defined as HCRU with a vitiligo diagnosis code associated with the claim. Additionally, treatment patterns among patients with vitiligo (overall and by line of vitiligo‐related therapy) were summarized.

Direct healthcare costs were assessed using the paid amounts recorded on adjudicated claims, including insurer and health plan payments as well as patient cost‐sharing in the form of copayment, deductible payments, and coinsurance. Costs for services provided under capitated arrangements were estimated using payment proxies based on paid claims at the procedure level. Total costs reported include medical, pharmaceutical, and procedure costs, as well as materials costs which are not reported separately. All direct costs were expressed in Japanese yen, converted to 2022 values assuming a 3% inflation rate, and calculated using the Medical Care component of the Consumer Price Index. Both unadjusted and adjusted direct costs were reported; adjustments were made for age, sex, index year, and pre‐index cost.

### 2.4 Statistical Analyses

For all outcomes, categorical variables are presented as absolute numbers and percentages, while continuous variables are summarized using means, medians, standard deviations and ranges. Unadjusted healthcare costs and HCRU were compared between the vitiligo cohort and matched non‐vitiligo controls, and within the vitiligo cohort by comorbid condition status, using chi‐square tests for categorical variables and analysis of variance (ANOVA)/*t*‐tests for continuous variables (significance threshold: *p* < 0.05). When comparing adjusted direct costs between patients with vitiligo with and without comorbid conditions, multivariate generalized linear models with a Gamma distribution and log link were used. Separate models were applied for inpatient, outpatient, pharmacy, and total direct healthcare costs, adjusting for patient age, sex, index year, and pre‐index cost. Mean cost ratios were reported for adjusted analyses. Treatment patterns within the vitiligo cohort were analyzed descriptively.

## 3 Results

### 3.1 Study Population

A total of 28 800 patients were included in the analysis (4800 in the vitiligo cohort; 24 000 controls) (Figure 1). Overall, baseline characteristics and demographics were balanced between the vitiligo cohort and controls (Table 1). Participants had a mean age of 40.2 years (more than half were aged 40 years or older), 47% were female, and the mean Charlson Comorbidity Index (CCI) score was 0.34 (indicating few or mild comorbidities). The most commonly reported comorbidities covered by the CCI were chronic pulmonary disease (9.9%), mild liver disease (5.5%), and peptic ulcer disease (3.8%). Psychiatric and autoimmune conditions were reported in 5.2% and 4.1% of participants, respectively, most commonly depression (2.8%), anxiety (2.0%), and atopic dermatitis (2.1%). Notably, autoimmune comorbidities were more common in the vitiligo cohort compared with controls (7.0% and 3.5%, respectively), in particular, atopic dermatitis (3.4% and 1.8%), alopecia (1.8% and 0.2%), and thyroid disease (1.1% and 0.6%).

**FIGURE 1:**
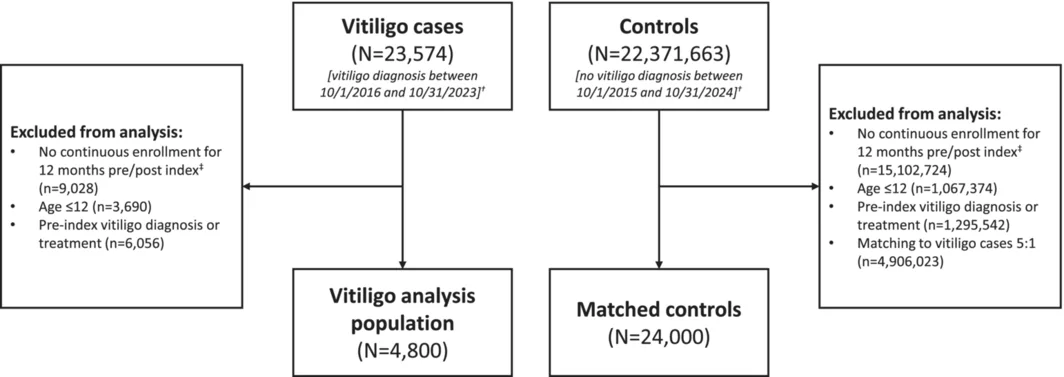
Study population disposition. ^†^Diagnosis‐based dates (including all index dates) were always assumed to occur on the 15th of each month; ^‡^start of enrollment was always assumed to occur on the 1st of each month and the end of enrollment on the 28th of each month.

**TABLE 1:** Baseline demographics and characteristics for matched vitiligo and control cohorts.

Characteristic	Case (*n* = 4800)	Control (*n* = 24 000)	Overall (*N* = 28 800)
**Age at index date, mean (SD), years**	40.2 (16.1)	40.2 (16.1)	40.2 (16.1)
Median (range)	42 (12–73)	42 (12–73)	42 (12–73)
**Age groups,** ***n*** **(%), years**			
12–14	412 (8.6)	2060 (8.6)	2472 (8.6)
15–18	329 (6.9)	1645 (6.9)	1974 (6.9)
19–29	579 (12.1)	2895 (12.1)	3474 (12.1)
30–39	829 (17.3)	4145 (17.3)	4974 (17.3)
40–49	1055 (22.0)	5275 (22.0)	6330 (22.0)
50–64	1389 (28.9)	6945 (28.9)	8334 (28.9)
65+	207 (4.3)	1035 (4.3)	1242 (4.3)
**Sex, female,** ***n*** **(%)**	2272 (47.3)	11 360 (47.3)	13 632 (47.3)
**BMI, mean (SD), kg/m** ^**2**^	22.6 (3.5)	23.1 (3.9)	23.0 (3.8)
CCI score, mean (SD)	0.39 (0.92)	0.33 (0.85)	0.34 (0.86)
**CCI comorbidities,** ***n*** **(%) [> 1%]**			
Chronic pulmonary disease	579 (12.1)	2284 (9.5)	2863 (9.9)
Mild liver disease	263 (5.5)	1331 (5.5)	1594 (5.5)
Peptic ulcer disease	219 (4.6)	870 (3.6)	1089 (3.8)
Any malignancy	121 (2.5)	483 (2.0)	604 (2.1)
Cerebrovascular disease	95 (2.0)	480 (2.0)	575 (2.0)
Congestive heart failure	71 (1.5)	375 (1.6)	446 (1.5)
Peripheral vascular disease	84 (1.8)	306 (1.3)	390 (1.4)
Diabetes with complications	48 (1.0)	292 (1.2)	340 (1.2)
**Psychiatric comorbidities** ^**a**^			
Any	269 (5.6%)	1233 (5.1%)	1502 (5.2%)
Depression	138 (2.9%)	682 (2.8%)	820 (2.8%)
Anxiety	119 (2.5%)	443 (1.8%)	562 (2.0%)
**Autoimmune comorbidities** ^**b**^			
Any	337 (7.0%)	849 (3.5%)	1186 (4.1%)
Diabetes	27 (0.6%)	148 (0.6%)	175 (0.6%)
Atopic dermatitis	164 (3.4%)	430 (1.8%)	594 (2.1%)
Alopecia areata	85 (1.8%)	41 (0.2%)	126 (0.4%)
Inflammatory bowel disease	24 (0.5%)	85 (0.4%)	109 (0.4%)
Autoimmune thyroid disease	53 (1.1%)	155 (0.6%)	208 (0.7%)

Abbreviations: BMI, body mass index; CCI, Charlson Comorbidity Index; SD, standard deviation.

^a^ Includes anxiety, sleep disturbance, depression, substance abuse or dependency, adjustment disorder, dysthymic disorder, panic disorder, alcohol abuse or dependency, suicidal ideation/attempts, bipolar disorder, sexual dysfunction, eating disorder, social phobia, agoraphobia, personality disorder, schizophrenia.

^b^ Includes autoimmune thyroid disease, psoriasis, diabetes, dermatomyositis, alopecia areata, systemic lupus erythematosus, rheumatoid arthritis, juvenile idiopathic arthritis, scleroderma, atopic dermatitis, pernicious anemia, Addison's, Sjogren's, inflammatory bowel disease.

### 3.2 Healthcare Costs and HCRU 12 Months Post Index in Patients With Vitiligo Versus Matched Non‐Vitiligo Controls

Overall, patients with vitiligo had significantly greater unadjusted direct healthcare costs than matched controls (¥84 766 vs. ¥55 011, *p* < 0.0001; Figure 2A). This was primarily driven by outpatient costs, dermatology costs, and vitiligo‐related procedure costs, which were significantly greater in patients with vitiligo compared with controls (all *p* ≤ 0.0005), whereas inpatient costs and prescription costs were similar between cohorts (Figure 2A). This trend was also seen in adjusted all‐cause direct costs (Figure 2B) and reflected in the unadjusted all‐cause HCRU pattern (Figure 2C), where significant increases (16.0%–26.2%; all *p* < 0.0001) in outpatient visits, dermatology visits, prescriptions, and vitiligo‐related procedures were seen in patients with vitiligo compared with matched controls. Of note, the increase in vitiligo‐related procedures was driven by an increased use of phototherapy (26.5% vs. 0.5% for patients with vitiligo and matched controls, respectively, *p* < 0.0001; Figure 2C). Emergency department visits and associated costs were negligible for both cohorts (HCRU: 0.5% [both cohorts]; mean costs: ¥161–178).

**FIGURE 2:**
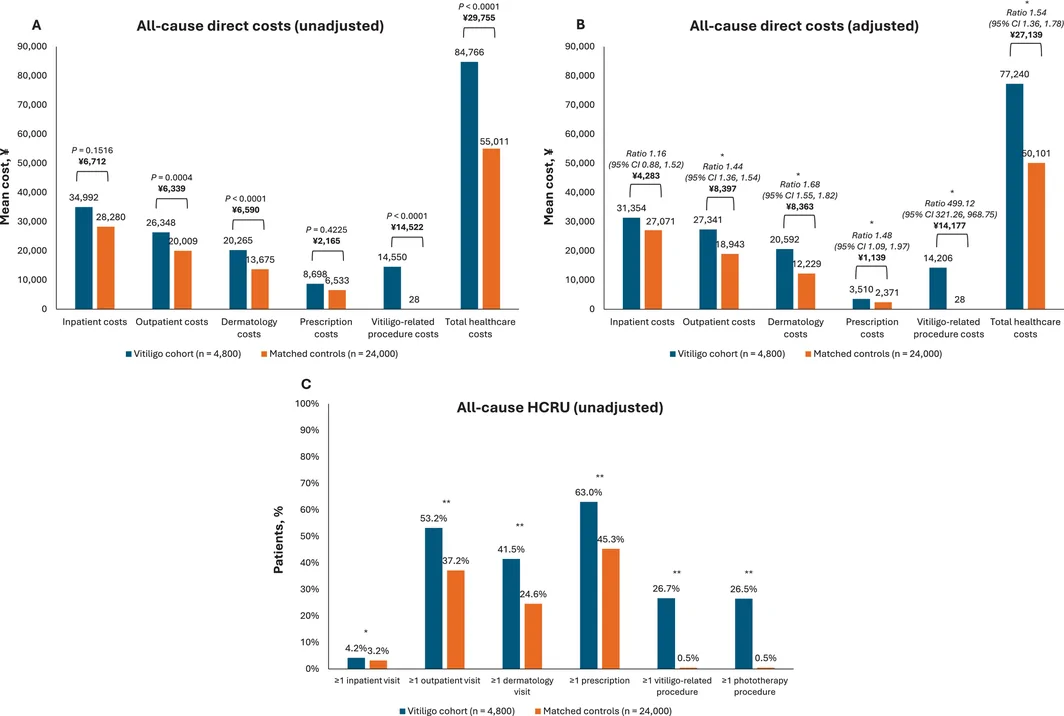
Mean (A) unadjusted and (B) adjusted all‐cause direct costs and (C) HCRU in patients with vitiligo and matched non‐vitiligo controls. *p < 0.05; **p < 0.0001. Adjustments were made based on patient age, sex, index year, and pre‐index costs in the specified category. Outpatient and dermatology costs are net of associated prescriptions and vitiligo‐related procedures. Vitiligo‐related procedures included dermabrasion, skin grafts, dermatological laser procedures, and phototherapy. CI, confidence interval; HCRU, healthcare resource utilization.

### 3.3 Healthcare Costs and HCRU 12 Months Post Index in Patients With Vitiligo and Psychiatric or Autoimmune Comorbidities Versus Those Without

#### 3.3.1 Comorbid Psychiatric Conditions

In patients with vitiligo (*n* = 4800), those with comorbid psychiatric conditions had significantly greater unadjusted all‐cause healthcare costs than those without a comorbid psychiatric condition 12 months post index (¥193 468 vs. ¥78 313, *p* < 0.0001; Figure 3A). This difference was driven by increased inpatient costs and prescription costs, with no significant differences observed for outpatient costs, dermatology costs, or vitiligo‐related procedure costs. Similar trends were seen with adjusted all‐cause costs (Figure 3B), though only numerical increases were observed. When all‐cause HCRU was analyzed, patients with vitiligo and with comorbid psychiatric conditions had increased percentage of prescriptions, outpatient, and dermatology visits compared to those without a comorbid psychiatric condition; inpatient, vitiligo‐related, and phototherapy visits were similar across all vitiligo patients (Figure 3C).

**FIGURE 3:**
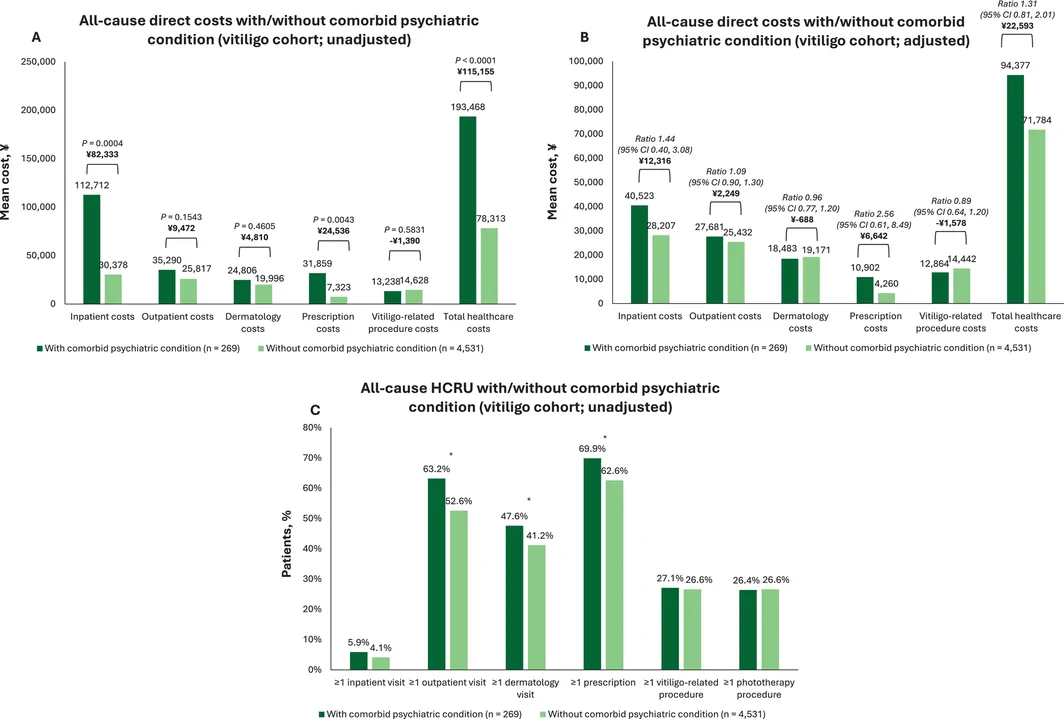
Mean (A) unadjusted and (B) adjusted all‐cause direct costs and (C) HCRU in patients with/without comorbid psychiatric^†^ conditions (vitiligo cohort). *p < 0.05. Adjustments were made based on patient age, sex, index year, and pre‐index costs in the specified category. Outpatient and dermatology costs are net of associated prescriptions and vitiligo‐related procedures. Vitiligo‐related procedures included dermabrasion, skin grafts, dermatological laser procedures, and phototherapy. ^†^Includes anxiety, sleep disturbance, depression, substance abuse or dependency, adjustment disorder, dysthymic disorder, panic disorder, alcohol abuse or dependency, suicidal ideation/attempts, bipolar disorder, sexual dysfunction, eating disorder, social phobia, agoraphobia, personality disorder, schizophrenia. CI, confidence interval; HCRU, healthcare resource utilization.

Patients with vitiligo and a comorbid psychiatric condition also had significantly greater unadjusted vitiligo‐related direct healthcare costs than those without a comorbid psychiatric condition (¥39 606 vs. ¥22 720, *p* = 0.0165 Figure 4A), which was driven by significant increases in outpatient costs, dermatology costs, and prescription costs. However, this trend was not observed for adjusted vitiligo‐related direct healthcare costs, where no significant differences were observed (Figure 4B). Vitiligo‐related HCRU was also similar for patients with or without a comorbid psychiatric condition; no differences > 1% were observed for any HCRU category assessed (Figure 4C).

**FIGURE 4:**
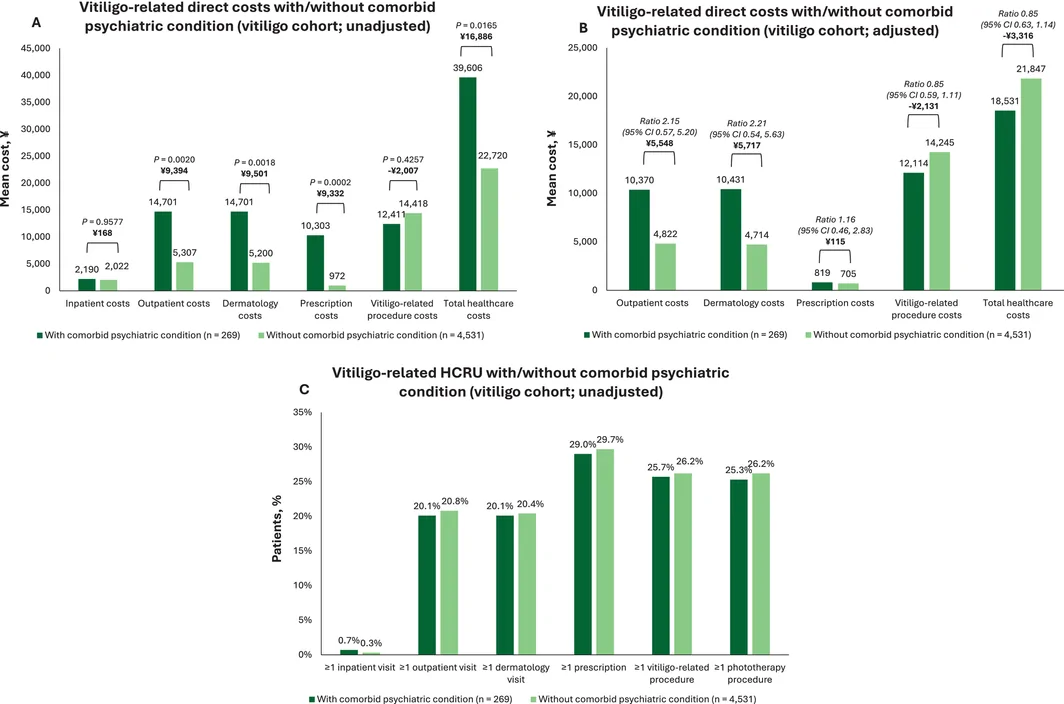
Mean (A) unadjusted and (B) adjusted vitiligo‐related direct costs and (C) HCRU in patients with/without comorbid psychiatric^†^ conditions (vitiligo cohort). Adjustments were made based on patient age, sex, index year, and pre‐index costs in the specified category. Outpatient and dermatology costs are net of associated prescriptions and vitiligo‐related procedures. Vitiligo‐related procedures included dermabrasion, skin grafts, dermatological laser procedures, and phototherapy. ^†^Includes anxiety, sleep disturbance, depression, substance abuse or dependency, adjustment disorder, dysthymic disorder, panic disorder, alcohol abuse or dependency, suicidal ideation/attempts, bipolar disorder, sexual dysfunction, eating disorder, social phobia, agoraphobia, personality disorder, schizophrenia. CI, confidence interval; HCRU, healthcare resource utilization.

#### 3.3.2 Comorbid Autoimmune Conditions

In patients with vitiligo (*n* = 4800), those with comorbid autoimmune conditions had a > 2‐fold greater unadjusted all‐cause direct healthcare costs than those without a comorbid autoimmune condition (¥185 179 vs. ¥77 184, *p* < 0.0001; Figure 5A). This difference was driven by significant increases in outpatient, dermatology, prescription, and vitiligo‐related procedure costs (Figure 5A). Similar results were seen for adjusted all‐cause healthcare costs (Figure 5B) and all‐cause HCRU (Figure 5C), with consistently greater costs and HCRU observed in patients with a comorbid autoimmune condition.

**FIGURE 5:**
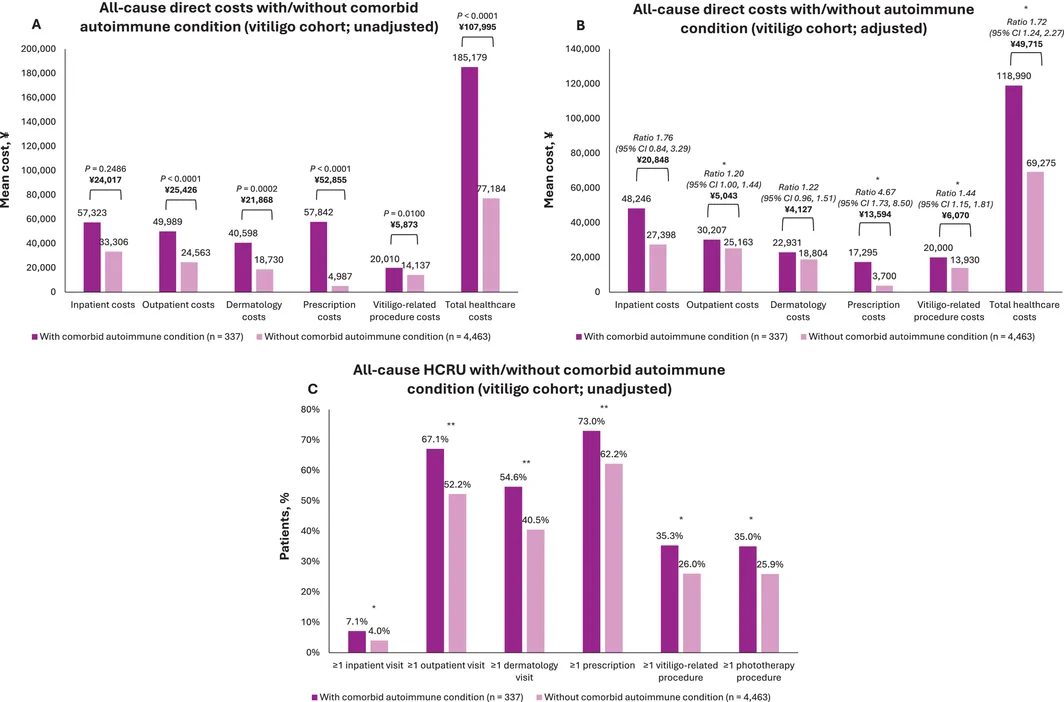
Mean (A) unadjusted and (B) adjusted all‐cause direct costs and (C) HCRU in patients with/without comorbid autoimmune^†^ conditions (vitiligo cohort). *p < 0.05; **p < 0.0001. Adjustments were made based on patient age, sex, index year, and pre‐index costs in the specified category. Outpatient and dermatology costs are net of associated prescriptions and vitiligo‐related procedures. Vitiligo‐related procedures included dermabrasion, skin grafts, dermatological laser procedures, and phototherapy. ^†^Includes autoimmune thyroid disease, psoriasis, diabetes, dermatomyositis, alopecia areata, systemic lupus erythematosus, rheumatoid arthritis, juvenile idiopathic arthritis, scleroderma, atopic dermatitis, pernicious anemia, Addison's, Sjogren's, inflammatory bowel disease. CI, confidence interval; HCRU, healthcare resource utilization.

Patients with vitiligo and a comorbid autoimmune condition also had significantly greater (> 2‐fold) unadjusted vitiligo‐related direct healthcare costs than those without a comorbid autoimmune condition (¥53 465 vs. ¥21 417, *p* < 0.0001; Figure 6A), and this trend was consistent across all cost categories (inpatient, outpatient, dermatology, prescription, and vitiligo‐related procedure costs; all *p* ≤ 0.02). Similar results were seen for adjusted vitiligo‐related direct healthcare costs (Figure 6B) and vitiligo‐related HCRU (Figure 6C), though the difference in outpatient and dermatology visits did not reach statistical significance.

**FIGURE 6:**
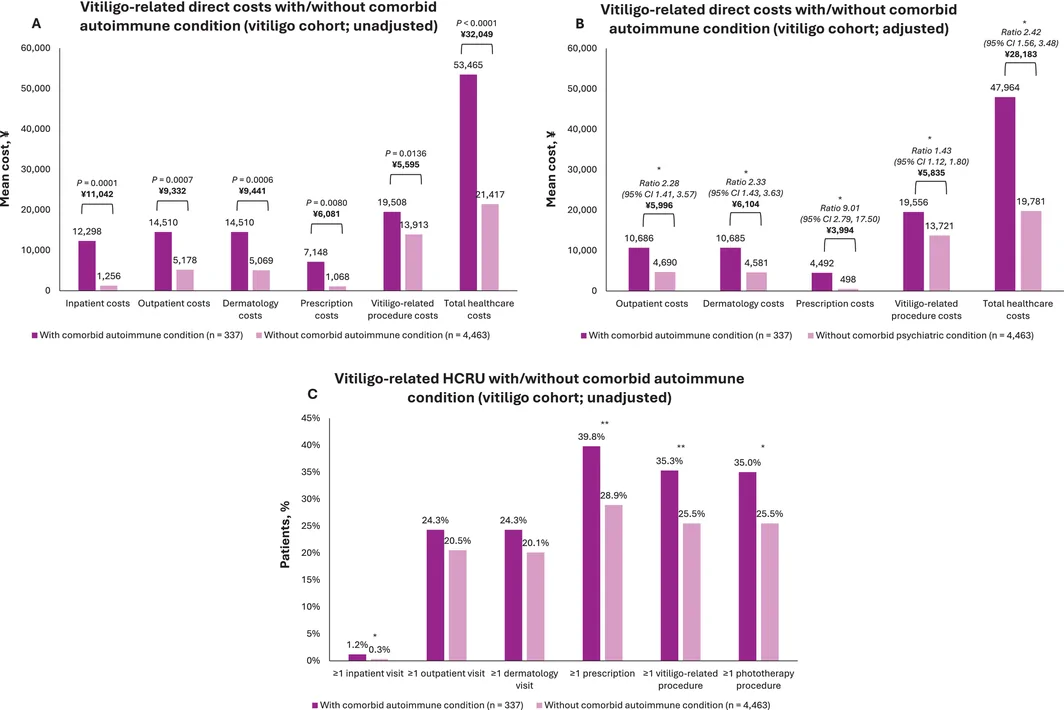
Mean (A) unadjusted and (B) adjusted all‐cause direct costs and (C) HCRU in patients with/without comorbid autoimmune† conditions (vitiligo cohort). *p < 0.05; **p < 0.0001. Adjustments were made based on patient age, sex, index year, and pre‐index costs in the specified category. Outpatient and dermatology costs are net of associated prescriptions and vitiligo‐related procedures. Vitiligo‐related procedures included dermabrasion, skin grafts, dermatological laser procedures, and phototherapy. ^†^Includes autoimmune thyroid disease, psoriasis, diabetes, dermatomyositis, alopecia areata, systemic lupus erythematosus, rheumatoid arthritis, juvenile idiopathic arthritis, scleroderma, atopic dermatitis, pernicious anemia, Addison's, Sjogren's, inflammatory bowel disease. CI, confidence interval; HCRU, healthcare resource utilization.

### 3.4 Treatment Patterns (Vitiligo‐Related)

Treatments for patients with vitiligo in the 12‐month post‐index period are summarized in Table 2. Overall, a large majority of patients (67.5%) did not receive any treatment for vitiligo in the 12 months following their diagnosis. Of the patients who did, most (*n* = 1187/1562 [76.0%]) received only phototherapy as their first‐line treatment, with the next most common first‐line treatments being a topical combination of corticosteroids with antibiotics (*n* = 106/1562 [6.8%]), topical corticosteroids (*n* = 100/1562 [6.4%]), or a topical calcineurin inhibitor (*n* = 53/1562 [3.4%]). No other first‐line treatment or combination of treatments were used in > 1% of patients overall. Out of the patients receiving treatment, only 2.8% received any form of second‐line treatment. Overall, 2.2% of all patients with vitiligo received a second‐line treatment.

**TABLE 2:** Treatment patterns in Japanese patients with vitiligo (12‐months post‐diagnosis), overall and with/without comorbid psychiatric or autoimmune conditions.

Treatment, *n* (%)	Overall (*n* = 4800)	Comorbid psychiatric condition^a^		Comorbid autoimmune condition^b^	
		With (*n* = 269)	Without (*n* = 4531)	With (*n* = 337)	Without (*n* = 4463)
Total					
Phototherapy/photochemotherapy	1274 (26.5)	71 (26.4)	1203 (26.6)	118 (35.0)	1156 (25.9)
Topical corticosteroids with antibiotics	171 (3.6)	12 (4.5)	159 (3.5)	17 (5.0)	154 (3.5)
Topical corticosteroids	163 (3.4)	9 (3.3)	154 (3.4)	11 (3.3)	152 (3.4)
TCI	83 (1.7)	3 (1.1)	80 (1.8)	10 (3.0)	73 (1.6)
OCS	23 (0.5)	0	23 (0.5)	4 (1.2)	19 (0.4)
OCS with antihistamines	18 (0.4)	2 (0.7)	16 (0.4)	1 (0.3)	17 (0.4)
Other^c^	8 (0.2)	3 (1.1)	5 (0.1)	1 (0.3)	7 (0.2)
**First‐line treatment**					
None	3238 (67.5)	179 (66.5)	3059 (67.5)	194 (57.6)	3044 (68.2)
Phototherapy	1187 (24.7)	66 (24.5)	1121 (24.7)	109 (32.3)	1078 (24.2)
Topical combination corticosteroids with antibiotics	106 (2.2)	9 (3.3)	97 (2.1)	9 (2.7)	97 (2.2)
Topical corticosteroids	100 (2.1)	4 (1.5)	96 (2.1)	4 (1.2)	96 (2.2)
TCI	53 (1.1)	3 (1.1)	50 (1.1)	7 (2.1)	46 (1.0)
Phototherapy, topical corticosteroids	26 (0.5)	3 (1.1)	23 (0.5)	3 (0.9)	23 (0.5)
Phototherapy, topical combination corticosteroids with antibiotics	26 (0.5)	1 (0.4)	25 (0.6)	3 (0.9)	23 (0.5)
Other^d^	64 (1.3)	4 (1.5)	60 (1.3)	8 (2.4)	56 (1.3)
**Second‐line treatment**					
None	4692 (97.8)	262 (97.4)	4430 (97.8)	324 (96.1)	4368 (97.9)
Phototherapy	48 (1.0)	3 (1.1)	45 (1.0)	5 (1.5)	43 (1.0)
Topical combination corticosteroids with antibiotics	14 (0.3)	1 (0.4)	13 (0.3)	1 (0.3)	13 (0.3)
Phototherapy, topical corticosteroids	8 (0.2)	0	8 (0.2)	1 (0.3)	7 (0.2)
Topical corticosteroids	10 (0.2)	1 (0.4)	9 (0.2)	3 (0.9)	7 (0.2)
Other^e^	28 (0.6)	2 (0.7)	26 (0.6)	3 (0.9)	25 (0.6)

Abbreviations: OCS, oral corticosteroids; TCI, topical calcineurin inhibitor.

^a^ Includes anxiety, sleep disturbance, depression, substance abuse or dependency, adjustment disorder, dysthymic disorder, panic disorder, alcohol abuse or dependency, suicidal ideation/attempts, bipolar disorder, sexual dysfunction, eating disorder, social phobia, agoraphobia, personality disorder, schizophrenia.

^b^ Includes autoimmune thyroid disease, psoriasis, diabetes, dermatomyositis, alopecia areata, systemic lupus erythematosus, rheumatoid arthritis, juvenile idiopathic arthritis, scleroderma, atopic dermatitis, pernicious anemia, Addison's, Sjogren's, inflammatory bowel disease.

^c^ Treatments reported at < 0.2% including systemic immunosuppressants, OCS with antihistamines, topical vitamin D with corticosteroids, dermabrasion, and autografts/tissue‐cultured grafts.

^d^ First‐line treatments reported at < 0.5% including dermabrasion, grafts, OCS (monotherapy or combination), phototherapy (in combination with TCI, topical corticosteroids, antibiotics, and/or a topical combination of vitamin D with corticosteroids), TCI (in combination with topical corticosteroids with or without antibiotics), topical corticosteroids with antibiotics, and a topical combination of vitamin D with corticosteroids.

^e^ Second‐line treatments reported at < 0.2% including dermabrasion, OCS (monotherapy or combination), TCI, TCI in combination with antibiotics and topical corticosteroids, and phototherapy in combination with TCI, topical corticosteroids, and/or antibiotics.

While the overall treatment pattern for patients with vitiligo and a comorbid psychiatric condition (at index) was similar to those without a comorbid psychiatric condition, including the use of first‐line phototherapy, more patients with a comorbid autoimmune condition received treatment compared to those without a comorbid autoimmune condition (42.4% vs. 31.8%, respectively); this difference was primarily driven by an increased use of phototherapy (32.3% vs. 24.2%).

## 4 Discussion

To our knowledge, this is the first retrospective claims database study to systematically assess the economic burden of vitiligo in Japan, as well as evaluate vitiligo‐related treatment patterns. Overall, the results clearly showed that patients with vitiligo had significantly greater healthcare costs and HCRU than matched controls in the 12 months following their initial diagnosis, primarily driven by outpatient, dermatology, prescription, and vitiligo‐related procedure costs. These findings are consistent with those reported in a similar retrospective, claims database study conducted in the US [3], which also showed a nearly 2‐fold increase in all‐cause direct healthcare costs in patients with vitiligo versus controls ($15 551 vs. $7735; *p* < 0.0001), highlighting the need for cost‐effective therapies for vitiligo.

This study additionally investigated whether the presence of a comorbid psychiatric or autoimmune condition in the vitiligo cohort impacted healthcare costs or HCRU. In patients with vitiligo and a comorbid psychiatric condition, unadjusted direct costs were significantly higher than the vitiligo‐only cohort. This aligns with previous database studies from the US in which patients with vitiligo with psychiatric comorbidities showed increased all‐cause costs and HCRU, driven primarily by inpatient visits, outpatient visits and prescription costs [3, 10]. In this study, unadjusted all‐cause HCRU was also significantly higher in vitiligo patients with a comorbid psychiatric condition with respect to outpatient visits, dermatology visits, and prescriptions, though there was no difference in inpatient visits. Vitiligo‐related HCRU was broadly balanced between patients with vitiligo with and without a comorbid psychiatric condition. This was mirrored in the US claims database study, where there was also no difference in inpatient or outpatient visits, or prescriptions between patients with vitiligo with and without a psychiatric condition [10]. Given the similarity in vitiligo‐related HCRU among patients with vitiligo with and without a comorbid psychiatric condition, the greater all‐cause HCRU observed in those with psychiatric conditions may be driven, at least in part, by the associated psychosocial burden.

Previous studies have reported that patients with vitiligo have a higher incidence and increased risk of developing comorbid autoimmune conditions [11]. In this study, patients with vitiligo and a comorbid autoimmune condition had a significant > 2‐fold increase in unadjusted all‐cause healthcare costs compared to patients with vitiligo without a comorbid autoimmune condition, reflected by consistent increases in adjusted costs, vitiligo‐related costs, and HCRU. Taken together these findings suggest that the presence of comorbid autoimmune conditions increased the economic burden experienced by patients with vitiligo. Overall, these findings indicate that the presence of a psychiatric or autoimmune comorbidity in patients with vitiligo can substantially exacerbate both the economic and HCRU burden of the condition.

In the context of findings from other vitiligo studies in different countries and/or regions [4, 13, 14], some notable differences in treatment practices were also apparent. While vitiligo remained untreated for at least a year after diagnosis in a large proportion of patients in recent retrospective US and UK database studies (49.9% and 60.8%, respectively) [4, 14], the proportion in Japan was even greater (67.5%). In addition, the type of treatment initially used in the United States was most commonly a topical/oral corticosteroid or topical calcineurin inhibitor (each used in 15%–25% of vitiligo cases), with a similar pattern reported in the United Kingdom, albeit with less use of oral corticosteroids (topical corticosteroids: 29%; topical calcineurin inhibitor: 12%; oral corticosteroid: 4%) [14]. Reports also suggest that phototherapy (monotherapy) is uncommon, being used/prescribed in 4%–9% of vitiligo cases in the United States and United Kingdom [4, 13]. First‐line treatment patterns were dramatically different in Japan, where approximately 25% of patients with vitiligo received phototherapy alone (representing 76.0% of patients who received any form of treatment in the first year after diagnosis) and corticosteroids and calcineurin inhibitors were used less frequently. However, it should be noted that Japanese claims data have a structural limitation in that prescriptions are not directly linked to a specific diagnosis code [15]. Therefore, topical corticosteroids or calcineurin inhibitors used in patients with vitiligo may also have been prescribed for other coexisting conditions, such as atopic dermatitis, for which these agents are likewise standard treatments in Japan [16, 17]. As a result, claims‐based analyses may misattribute some of these therapies as vitiligo‐related treatment, which would tend to underestimate the proportion of patients receiving no vitiligo‐related treatment after diagnosis, consistent with prior acknowledgments in studies using the JMDC database [18].

Furthermore, in this study, patients in the vitiligo cohort were excluded from the dataset if they had evidence of vitiligo‐related treatment during the 12‐month pre‐index period. This ensures that treatments received in the post‐index period are for vitiligo only, which allows for analysis of treatment patterns; however, many common autoimmune comorbidities share similar treatments and removing these patients may lead to underestimation of comorbidity prevalence. This may explain why the prevalence of comorbidities such as atopic dermatitis and alopecia areata were much lower than those recently reported in a cross‐sectional study of patients with vitiligo in the JMDC [18]. Whether these differences are due to variations in disease severity/symptoms, treatment guidelines, or the patient/physician preferences in Japan, and whether these differences may impact treatment outcomes, is unclear due to the limited data available in claims databases on symptoms or disease severity. However, future studies warrant an investigation into these aspects using different sources of data to inform resource allocation and potentially improve the efficiency of health service utilization for vitiligo.

As with all studies, there are strengths and limitations that should be considered when interpreting the data. Strengths include the use of a large, geographically, and socioeconomically diverse commercial insurance database. Limitations include the incomplete nature of data recorded in administrative claims databases, which can have limited information on vitiligo‐related symptoms, safety outcomes, or treatments (e.g., they do not account for over‐the‐counter medications, point‐of‐care treatment, out‐of‐pocket prescription expenses, or other self‐management approaches, nor do they cover experimental and investigational therapies). As a result, this study cannot fully account for differences in disease severity that may influence treatment choices and outcomes and may not be fully generalizable to a Japanese population. Additionally, the dataset did not allow for differentiation between segmental and nonsegmental vitiligo. However, given that approximately 85% of vitiligo cases are nonsegmental, most cases in this cohort are likely to represent the nonsegmental subtype [19]. Another limitation is the nonrandomized selection process for the vitiligo cohort and the requirement for 12 months of continuous enrollment following the index date, which together could lead to a patient population more representative of a commercially insured and clinically stable vitiligo population than the overall Japanese vitiligo population. This also introduces immortal time bias, potentially limiting generalizability and underestimating early HCRU and costs. However, considering the large, diverse, dataset provided by the JMDC, these factors would have minimal impact on the overall outcomes and that the analysis presented here provides a robust picture of the current vitiligo treatment landscape and economic burden in Japan.

The results of this retrospective, claims database study showed that patients with vitiligo face a marked and significantly higher economic and HCRU burden than those without vitiligo, and that this burden is exacerbated by the presence of comorbid psychiatric and autoimmune conditions. Treatment pattern analysis also showed that approximately 70% of all Japanese patients did not receive treatment for vitiligo in the 12 months following diagnosis, and for those who did receive treatment, almost all exclusively received phototherapy. These findings highlight the current unmet needs, costs and challenges faced by patients and payers alike in the management of vitiligo.

## Funding

This work was supported by AbbVie.

## Ethics Statement

As this was a retrospective study using only anonymized data from the JMDC, no ethics committee/Institutional Review Board approval or informed patient consent was required.

## Conflicts of Interest

N.O. reports receiving honorarium for speaking from UCB Japan and is currently serving as the secretary general of the Japanese Society of Vitiligo and is an Editorial Board member of Journal of Dermatology. To minimize bias, they were excluded from all editorial decision‐making related to the acceptance of this article for publication. K.A.H., M.T., and CI are salaried full‐time employees of AbbVie and may hold AbbVie stocks. A.M.S. is an employee of AbbVie, may hold stocks in AbbVie and is a co‐inventor on AbbVie patents. DM, AR, DB, and H.S. are employees of Genesis Research Group and were contracted to conduct the data analyses. T.S. has served as a consultant for AbbVie, Pfizer, J‐TEC and Pola; and is currently serving as the president of the Japanese Society of Vitiligo and is an Editorial Board member of Journal of Dermatology. To minimize bias, they were excluded from all editorial decision‐making related to the acceptance of this article for publication.

## Data Availability

The data that support the findings of this study are available on request from the corresponding author. The data are not publicly available due to privacy or ethical restrictions.
